# A Diagnostic Cascade Triggered by Physical Examination: Uncovering Asymptomatic Left Ventricular Dysfunction and Critical Carotid Disease in a Hypertensive Patient

**DOI:** 10.7759/cureus.111411

**Published:** 2026-06-24

**Authors:** Xiuhong Lyu

**Affiliations:** 1 Hospital Medicine, Brown University Health, Providence, USA

**Keywords:** asymptomatic left ventricular systolic dysfunction, guideline directed medical therapy, physical examination, primary hypertension, systolic heart failure

## Abstract

Asymptomatic left ventricular systolic dysfunction (ALVSD) is a relatively uncommon condition. When left untreated, it carries the risk of progression to overt heart failure and adverse clinical outcomes. Here we present a case of a 60-year-old man with hypertension in whom severe left ventricular systolic dysfunction was identified in the absence of typical heart failure symptoms - uncovered through careful physical examination and directed diagnostic workup. The diagnostic process illustrates the value of thorough bedside assessment in revealing subclinical findings that might otherwise go undetected. Early recognition of severe left ventricular dysfunction at a pre-symptomatic stage allowed for the timely initiation of aggressive guideline-directed medical therapy (GDMT). Left ventricular ejection fraction improved from 30-35% to 44% within four months of treatment, and the patient remained free from heart failure hospitalization throughout the follow-up period. This case underscores the enduring importance of comprehensive physical examination - the most fundamental yet powerful tool available in clinical practice - in generating and refining the differential diagnosis. It also demonstrates how a carefully performed examination during a routine ambulatory encounter can profoundly alter the trajectory of patient care, particularly in resource-limited settings where access to advanced diagnostic testing may be constrained.

## Introduction

Asymptomatic left ventricular systolic dysfunction (ALVSD) is defined as depressed left ventricular systolic function, specifically, a left ventricular ejection fraction (LVEF) below 55% in the absence of heart failure symptoms, and is classified as stage B heart failure according to the American College of Cardiology/American Heart Association staging system [1]. Reported prevalence ranges from 1% to 8% [2-4].

Because ALVSD is, by definition, clinically asymptomatic, it is rarely identified through symptom-driven evaluation. Detection typically occurs incidentally - through physical examination findings such as a displaced point of maximal impulse or an S3 gallop, through echocardiography performed for an unrelated indication, or increasingly through opportunistic screening in high-risk populations, including patients with a longstanding hypertension, diabetes, or prior cardiotoxic exposure. Hypertension is one of the modifiable risk factors for ALVSD: chronic afterload elevation promotes progressive myocardial remodeling, beginning with compensatory and concentric hypertrophy and culminating in systolic dysfunction once decompensating. This pathophysiologic continuum underscores why hypertensive patients warrant a lower threshold for structural cardiac evaluation even in the absence of cardiac symptoms.

When left untreated, ALVSD can progress to overt heart failure with significant adverse clinical consequences [3]. Data from the Framingham Study demonstrated that patients with ALVSD faced a nearly fourfold increase in mortality risk compared to those with a normal LVEF [5]. Early detection is therefore of paramount clinical importance: initiation of guideline-directed medical therapy (GDMT) has been shown to halt or reverse adverse remodeling, improve ejection fraction, and reduce progression to symptomatic heart failure and mortality when initiated at the Stage B phase [6,7].

We present a case of a patient with longstanding hypertension who was initially evaluated in the ambulatory setting for a two-year history of nonspecific dizziness, later found to have ALVSD, which was deemed not to be the etiology of his dizziness. The diagnostic process illustrates the value of thorough bedside assessment in revealing subclinical findings that might otherwise go undetected, and demonstrates how incidental identification of ALVSD - particularly in a patient with hypertension, a well-established risk factor - can profoundly alter the trajectory of patient care through early initiation of disease-modifying therapy.

## Case presentation

A 60-year-old Portuguese-speaking man with a history of hypertension presented to the clinic with dizziness for two years. He described the symptoms as intermittent, occurring predominantly with positional changes and without an identifiable precipitating factor at onset. He took no medication.

On initial presentation, vital signs were notable for a blood pressure of 200/120 mmHg on the left arm, heart rate of 82 beats per minute, temperature of 98.1°F, and respiratory rate of 16 breaths per minute. Cardiovascular examination revealed normal S1, S2, regular rate and rhythm, and there were no murmurs, gallops, or rubs. Neurological examination was unremarkable. The initial impression was that his dizziness was due to uncontrolled hypertension with possible concurrent orthostatic hypotension. He received 0.1 mg of clonidine in the office, which reduced his blood pressure to 162/84 mmHg, and was subsequently started on amlodipine 5 mg once daily.

At a follow-up visit, the patient reported no improvement in dizziness and denied any focal weakness or sensory disturbance. Blood pressure measured 180/76 mmHg. Cardiac auscultation on this visit revealed a regularly irregular heartbeat, and a loud bruit was appreciated over the right carotid artery. There were no pulmonary crackles, jugular venous distension, or lower extremity edema. Point-of-care electrocardiography demonstrated sinus rhythm with frequent premature ventricular contractions (PVCs) in a near-bigeminal pattern (Figure 1).

**Figure 1:**
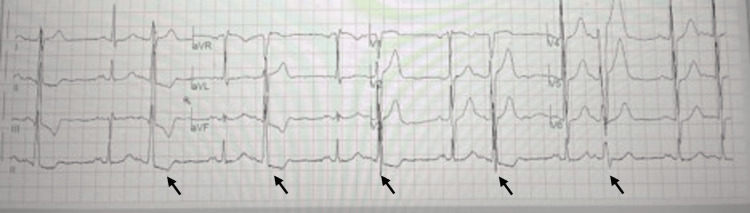
12-lead electrocardiogram during the follow-up visit Point-of-care 12-lead electrocardiogram in the ambulatory clinic showed sinus rhythm with bigeminal premature ventricular contractions (with arrow).

Notably, the previous EKG in the system showed a normal sinus rhythm (Figure 2).

**Figure 2:**
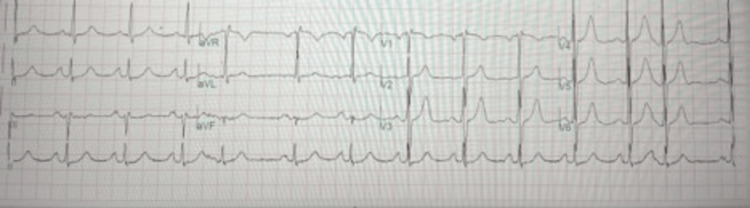
Previous electrocardiogram for comparison The previous ECG showed a normal sinus rhythm.

Given these findings, a cardiac etiology - specifically arrhythmia - was added to the differential diagnosis for his dizziness. More history was taken, which revealed that the patient had no chest pain/dyspnea/exertional dyspnea/PND/orthopnea/lower extremity edema. Moreover, a Holter monitor was arranged. In light of persistent hypertension and frequent PVCs, an echocardiogram was also ordered. Two weeks later, echocardiography revealed a mildly dilated left ventricle with an estimated ejection fraction of 30-35% and moderate global hypokinesis. Carotid duplex ultrasound demonstrated total occlusion of the left internal carotid artery and critical stenosis of the right internal carotid artery. Further blood work demonstrated BNP 1109 pg/ml. LDL 127 mg/dL. Hemoglobin A1 was within the normal limit. Aspirin 81 mg daily and atorvastatin 40 mg daily were initiated. Concurrently, Holter monitor results revealed there was one run of wide complex tachycardia consistent with ventricular tachycardia totaling 18 beats at a rate of up to 147 beats per minute. Unexpectedly, atrial fibrillation was detected on day 3 with an episode of 12 minutes and 52 seconds. Overall burden of atrial fibrillation was 0.41%. There was a high degree of atrial ectopy with a burden of 12.65%. The patient was started on apixaban 5 mg twice daily and lisinopril 2.5 mg daily; amlodipine was discontinued. He was also started on Metoprolol succinate 25 mg daily and spironolactone 25 mg daily.

The patient was seen by a cardiologist three months later. In the interim, his medications were up-titrated at the ambulatory clinic. At his cardiology visit, empagliflozin 10 mg daily was initiated, lisinopril was discontinued, and sacubitril-valsartan 24 mg/26 mg twice daily (Entresto) was started. Metoprolol succinate was up-titrated to 200 mg daily. Pharmacological SPECT nuclear stress testing identified a small area of mixed scar and ischemia in the apical-mid inferior wall. Cardiac CT demonstrated aortic and coronary calcification. Left heart catheterization revealed only mild-to-moderate nonobstructive coronary atherosclerosis, for which continued medical management was recommended.

A repeat echocardiogram obtained four months after the initiation of guideline-directed medical therapy (GDMT) demonstrated improvement in left ventricular ejection fraction from 30-35% to 44%. His follow-up BP was 140/82 mmHg. See Table 1 for comparisons of echocardiogram parameters before and after four-month GDMT.

**Table 1:** Comparison of Initial and Follow-Up Transthoracic Echocardiogram (TTE) Findings

Parameter	Initial TTE	Follow-Up TTE (4 months post-GDMT)
Left ventricular ejection fraction (LVEF)	30–35%	44%
Left ventricle	Mildly dilated with moderate global hypokinesis	Mildly dilated with mild to moderate global hypokinesis
Left atrium	Moderately dilated	Severely dilated
Right atrium	Moderately dilated	Severe dilated
Right ventricle	Severely dilated	Severely dilated
Aortic valve	Trace regurgitation	Mildly calcified, no stenosis
Mitral valve	Moderate regurgitation	Moderate regurgitation
Tricuspid valve	Moderate regurgitation	1+ (mild) regurgitation
RVSP / PASP	61 mmHg (markedly increased)	23 mmHg

The patient also underwent right carotid endarterectomy for critical right internal carotid artery stenosis without complication. Notably, his dizziness remained unchanged following the procedure and the above treatment. He was never hospitalized for heart failure.

## Discussion

Early detection of asymptomatic left ventricular dysfunction is clinically meaningful, as timely initiation of pharmacological therapy has been shown to slow disease progression and reduce the risk of advancement to overt heart failure, thereby improving outcomes in this population [6,7]. Nevertheless, routine echocardiographic screening for ALVSD is not currently recommended, and its cost-effectiveness remains uncertain [8].

Several investigational tools have been proposed as potential aids in identifying patients with ALVSD. In the review article by Jaskanwal et al. [9], three approaches were highlighted: serum B-type natriuretic peptide (BNP) [10], artificial intelligence-assisted analysis of the standard electrocardiogram [11], and non-invasive assessment of peripheral endothelial dysfunction (PED) [12]. Each, however, carries important limitations. The cost-effectiveness of BNP-based screening has yet to be established. The clinical applicability of AI-assisted ECG algorithms is constrained by an incomplete understanding of which specific electrocardiographic abnormalities these models detect and how reliably they correlate with ventricular dysfunction. The precise mechanistic relationship between PED and ALVSD remains to be fully elucidated. This diagnostic gap becomes particularly consequential in day-to-day clinical practice, where the inherently asymptomatic nature of the condition means that affected patients rarely present with complaints that would prompt targeted cardiac evaluation.

In the case presented here, one might argue that his initial presentation of the two-year history of dizziness could represent an early manifestation of heart failure rather than truly asymptomatic left ventricular dysfunction. The author does not favor this interpretation. While dizziness is a recognized symptom in patients with overt heart failure - reflecting impaired end-organ perfusion in the setting of reduced cardiac output - it is not, in isolation, a symptom that reliably directs the differential diagnosis toward heart failure. In the absence of accompanying symptoms such as dyspnea, paroxysmal nocturnal dyspnea, or lower extremity edema, dizziness alone would not typically raise clinical suspicion for ventricular dysfunction, as this case illustrated.

The diagnostic cascade in this case was triggered by two physical examination findings: a regularly irregular heartbeat and a right carotid bruit. The irregular rhythm prompted point-of-care electrocardiography, which revealed frequent PVCs in a bigeminal pattern. At that juncture, left ventricular systolic dysfunction remained low on the differential, given the absence of any clinical signs or symptoms of heart failure. Nevertheless, several questions naturally arose: Could frequent PVCs be responsible for two years of dizziness? Could the carotid bruit reflect hemodynamically significant stenosis contributing to cerebral hypoperfusion? And if the PVC burden were substantial, what might its cumulative effect be on ventricular function over time? These considerations prompted Holter monitoring, echocardiography, and carotid duplex ultrasound studies that collectively revealed atrial fibrillation, left ventricular systolic dysfunction with an EF of 30-35%, moderate global hypokinesis, and bilateral internal carotid artery stenosis.

Given the only mild-to-moderate nonobstructive coronary atherosclerosis findings in heart catheterization, we favored non-ischemic cardiomyopathy as the general etiology of left ventricle dysfunction. Specifically, hypertensive cardiomyopathy was favored as the most likely etiology, given the patient's severely uncontrolled blood pressure (200/120 mmHg) at presentation. The absence of LV hypertrophy does not exclude this diagnosis, as systolic dysfunction can occur without a sustained hypertrophic phase in cases of severe or abrupt hypertensive injury. Dilated cardiomyopathy was also on the differential diagnosis given the improvement of LVEF after treatment. The low PVC burden on Holter monitoring argued against a primary arrhythmia-mediated cardiomyopathy, and the substantial EF improvement with guideline-directed medical therapy supports a partially reversible hypertensive mechanism. Notably, several alternative etiologies warranted consideration. Hypertrophic cardiomyopathy and its dilated-phase variant were possible but less likely in the absence of left ventricular hypertrophy on echocardiography, no LVH voltage criteria or conduction abnormalities on ECG, and no family history of HCM or sudden cardiac death. Cardiac amyloidosis was considered less likely as well, given the absence of LV hypertrophy and normal ECG voltage, though strain imaging was not performed. Fabry disease was considered low probability in this 60-year-old male, given the absence of LV hypertrophy, family history, or extracardiac findings; however, one must notice that enzyme testing was not performed in the current study. Furthermore, whether premature ventricular contractions, newly detected atrial fibrillation, or uncontrolled hypertension should independently lower the threshold for echocardiographic assessment in asymptomatic patients remains to be determined, as this case alone provides insufficient data to support such a recommendation. Notably, this patient’s dizziness persisted after optimizing cardiac function and right carotid endarterectomy, suggesting an alternative, such as psychogenic dizziness - and as yet unidentified - etiology accounts for his dizziness, reinforcing the necessity of subsequent referral to Neurology rather than attributing his symptoms to either his carotid disease or his cardiomyopathy.

This case illustrates the enduring value of comprehensive physical examination in generating and refining the differential diagnosis. It also serves as a compelling example of how a carefully performed bedside assessment during a routine ambulatory encounter can profoundly alter the course of patient management, particularly in resource-limited populations. As medicine has grown increasingly reliant on imaging and ancillary testing, with mounting time pressures in clinical ambulatory practice, the primacy of physical examination has gradually eroded. Yet as this case reminds us, no technology can substitute for what was uncovered at the bedside. Seeing, listening to, and examining the patient must always form the foundation of clinical diagnosis - an irreplaceable first step that no laboratory value or imaging study can fully replicate.

This case has several limitations. The etiology of the patient's dizziness remains undetermined, and he was referred to Neurology for further evaluation. Similarly, the precise etiology of his left ventricular systolic dysfunction cannot be established with certainty; while a hypertensive cardiomyopathy was favored based on his clinical history and the absence of features supporting alternative diagnoses, confirmatory testing such as cardiac MRI, endomyocardial biopsy, or genetic testing was not performed. 

## Conclusions

This case illustrates how a routine ambulatory encounter, guided by careful observation and auscultation alone, triggered a diagnostic workup that uncovered clinically significant and otherwise silent left ventricular systolic dysfunction - a finding that fundamentally altered the patient's management and long-term prognosis. Although this patient's presenting complaint of chronic dizziness was ultimately determined to be largely unrelated to his cardiac findings, this case nonetheless underscores the value of a thorough bedside assessment in identifying findings that warrant further investigation, even when the presenting complaint appears unrelated to the eventual diagnosis. As time pressures and increasing technological reliance continue to reshape clinical practice, especially in the ambulatory setting, this case reminds us to preserve the primacy of bedside assessment. Nothing can replace the attentive clinician who takes the time to truly examine the patient.
